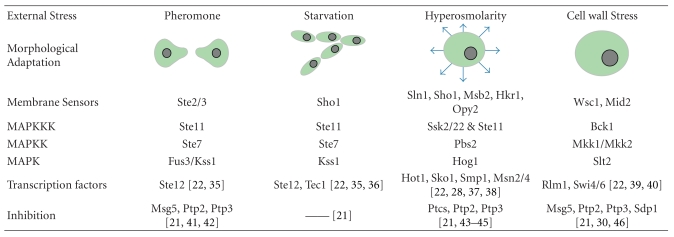# The Dynamical Systems Properties of the HOG Signaling Cascade

**DOI:** 10.1155/2011/930940

**Published:** 2011-02-07

**Authors:** Agnès Miermont, Jannis Uhlendorf, Megan McClean, Pascal Hersen

**Affiliations:** ^1^Laboratoire Matière et Systèmes Complexes, UMR7057, CNRS and Université Paris Diderot, 10 rue Alice Domon et Léonie Duquet, 75013 Paris, France; ^2^Contraintes Research Group, Institut National de Recherche en Informatique et en Automatique, INRIA Paris—Rocquencourt, France; ^3^Lewis-Sigler Institute for Integrative Genomics, Princeton University, Princeton, NJ 08540, USA; ^4^The Mechanobiology Institute, National University of Singapore, 5A Engineering Drive 1, Singapore 117411, Singapore

## Abstract

The High Osmolarity Glycerol (HOG) MAP kinase pathway in the budding yeast *Saccharomyces cerevisiae* is one of the best characterized model signaling pathways. The pathway processes external signals of increased osmolarity into appropriate physiological responses within the yeast cell. Recent advances in microfluidic technology coupled with quantitative modeling, and techniques from reverse systems engineering have allowed yet further insight into this already well-understood pathway. These new techniques are essential for understanding the dynamical processes at play when cells process external stimuli into biological responses. They are widely applicable to other signaling pathways of interest. Here, we review the recent advances brought by these approaches in the context of understanding the dynamics of the HOG pathway signaling.

## 1. Introduction

Living organisms have evolved specialized biochemical pathways to cope with stressful, often changing environments. Even in simple cells such as yeast, thousands of specialized sets of sensing and signaling proteins form modules used to monitor and adapt to the environmental state and its variations. Such modules can be insulated or, on the contrary, connected to one another. Whereas insulation allows for robust and sensitive response, the interconnection of modules allows for higher-level behavior such as multiple input sensing and decision making through cross-talk [1]. For a given stimulus, the biochemical components of the different modules that play a role in the cellular response are usually well described in the literature. Their biological functions and interactions are known in detail, especially in model organisms such as the budding yeast. This knowledge comes from decades of complex, tedious, and elegant experiments. Genetic techniques such as gene deletion, mutation, and overexpression have been used to infer the connection patterns between proteins and the architectures of many modular functions. Biochemical assays provided crucial information on protein phosphorylation and kinase activity. Microarrays revealed the role of these modules in determining global gene expression.

Signaling pathways are naturally dynamic [2] in that cells must respond to external signals in a timely manner, and indeed, the cellular response is often affected by the temporal properties of the external signal. In addition, the internal dynamics and timing of events in the signaling pathway determine the cellular response. These internal dynamics determine the information flow, allowing cells to process and convey information from a sensory input to a specific protein in charge of orchestrating the cellular response [3]. Until recently, experimental techniques have been limited such that most studies have examined the response of a signaling pathway to a stationary stimulus. Accordingly, adaptation and cellular responses to environmental cues were usually studied only with respect to the magnitude of the stimulus without seriously taking into account dynamical aspects. Identification of the components of a signaling pathway through the techniques mentioned above, combined with studies of simple stationary stimuli, is not enough to understand the dynamics or systems-level properties of a complex biological network. 

With the emergence of systems biology, there has been an important paradigm shift, and it is becoming increasingly clear that the temporal variations of stimulatory inputs can be directly sensed by cells [5] and that studying cells in time-variable environments is a powerful way to determine signaling pathway architecture and to understand how they process information [6, 7]. Experimental microfluidics-based strategies have matured to allow for excellent control of the cellular environment both in time and space [8, 9]. This technology coupled with genetic engineering to fluorescently tagged proteins allows for real-time observation of the system's response using fluorescence microscopy. Finally, quantitative real-time measurements form the basis for the development of mathematical models and the use of signal analysis tools, such as reverse engineering, to model the dynamical aspects of signaling pathways [10]. These models in turn provide testable experimental predictions.

This review describes the recent strategies that have been developed to assess quantitatively the dynamics of the canonical HOG MAP kinase (MAPK) pathway in the yeast, *Saccharomyces cerevisiae*. We shall first briefly review the key characteristics of the organization of the HOG pathway. We then discuss the novel experimental and modeling tools [10, 11, 16–18] that are allowing new insights into the pathway's dynamics and systems-level behavior.

## 2. MAPK Cascades in Yeast

**Figure 1:**
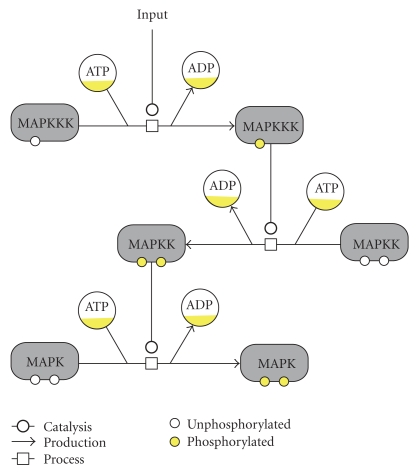
The canonical structure of a MAPK cascade. We used the Systems Biology Graphical Notation (SBGN) [4] to represent the interactions between the MAP Kinases. Activations of MAPK occur through enzymatic phosphorylation and ATP consumption. Interactions with other components and in particular with phosphatases are not shown. In the case of the HOG pathway in yeast, dual phosphorylation of the final MAPK (Hog1p) occurs within a few minutes after an hyper-osmotic stimulus.

Among signaling pathways, the Mitogen Activated Protein Kinases (MAPK) family has received considerable attention. MAPK pathways are very well conserved from yeasts to mammals [19–21] and several comprehensive reviews are available in the recent literature [22, 23]. MAP Kinase pathways are involved in many cellular processes such as stress response, the regulation of differentiation and proliferation. These pathways contain a canonical module of three protein kinases that act in series (Figure 1). Upon phosphorylation by an upstream protein, a MAP kinase kinase kinase (MAPKKK) phosphorylates a MAP kinase kinase (MAPKK) on conserved serine and threonine residues, which in turn phosphorylates a MAP kinase (MAPK) on a threonine (sometimes serine) and a tyrosine residue located adjacent to each other and separated by a single amino acid (Thr/Ser-X-Tyr). This dual phosphorylation site is located in the activation loop of the catalytic domain and its dual phosphorylation is needed for activation of the MAP kinase. 

**Figure 2:**
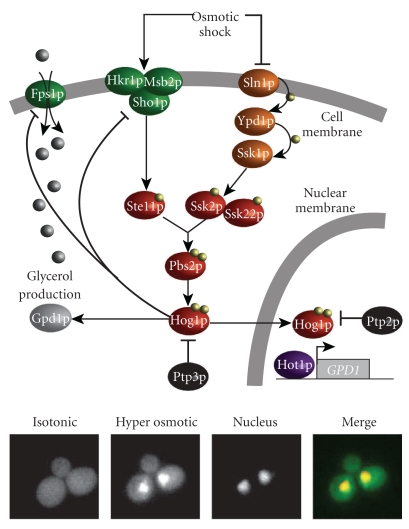
The HOG pathway. View of the main molecular actors involved in the hyperosmotic glycerol pathway (see text for more details). Two branches led by Sho1p and Sln1p are sensitive to high osmolarity and lead to the activation of Pbs2p and Hog1p after a hyperosmotic shock. Hog1p has both a cytoplasmic and a nuclear role, with different timescales, that correspond to a fast non transcriptional response and a longer response involving transcription when dealing with strong hyper osmotic shock. The yeast pictures at the bottom show nuclear localization of Hog1p tagged by GFP after a moderate hyper-osmotic shock (Sorbitol, 1 M). Colocalization with the nucleus is seen on the overlay pictures between the GFP channel (Hog1p) and the RFP channel (Htb2p). Note that localization is transient and reversible if the cell is put back into isotonic conditions.

There are five MAPK modules in yeast (Table 1) [22]. The hyperosmotic glycerol (HOG) pathway is activated in response to a hyperosmotic stress [24–28]. The Cell Wall Integrity (CWI) module controls the cell wall integrity and is triggered in response to numerous stresses including cell wall deterioration, temperature shifts, and hypo osmotic shocks [29–31]. The pheromone pathway [32, 33] controls the mating response which involves an important morphological deformation of yeast cells. Finally, the filamentous growth pathway [33, 34] and the sporulation pathway [22] control the response to starvation for haploid and diploid cells although the sporulation pathway is not as well known as the other four MAPK pathways. Only its MAPK has been identified in diploid cells (Smk1p), and it is thought to drive the spore cell wall assembly [22]. Though they share numerous components, the five MAPK pathways of the yeast *Saccharomyces cerevisiae* are tightly regulated by cross-talk and mutual inhibition which permit faithful signaling, adaptation to their environment, and regulation of growth and morphogenesis [22]. Among these MAPK pathways, the HOG pathway (Figure 2) is particularly well suited to study signaling dynamics, since it can be reliably activated through increasing the osmolarity of the environment. 

## 3. The HOG MAPK Signaling Pathway

Water homeostasis is fundamental for life. In nature, the environment can vary rapidly from isotonic to hyper or hypo osmotic conditions, and yeast cells have to adapt quickly [23, 47]. The first response after a hyperosmotic shock is the rapid loss in few seconds of cell volume due to water efflux and the activation of membrane sensory receptors followed by the activation of the HOG pathway which is completed after a few minutes (Figure 2) [11, 48]. Two distinct branches of the pathway detect changes in osmolarity and activate the pathway. These branches converge at the level of the MAPKK Pbs2p. The first branch is referred to as the SHO1 branch [23, 49], while the second is referred to as the SLN1 branch [50, 51].

Sln1p negatively regulates the HOG signaling pathway and deletion of *SLN1* is lethal due to pathway overactivation. This lethality is suppressed by knocking out any of the downstream components *SSK1*, *SSK2/SSK22*, *PBS2*, or *HOG1*. Sln1p contains two transmembrane domains, a histidine kinase domain and a receiver sequence. Sln1p autophosphorylates on its histidine kinase domain. The phosphate group is then transferred to its receiver domain, then to Ypd1p and finally to Ssk1p. This set of three proteins forms a phosphorelay [50, 51], a very common signaling motif in prokaryotes [51], but rare in eukaryotic cells such as yeast. The phosphorylated form of Ssk1p is inactive and the downstream MAPK pathway is usually not activated. However, after a hyperosmotic stress, Sln1p is inactivated by an unknown mechanism (though it has been proposed that Sln1p is sensitive to membrane tension [23, 52]) leading to the inactivation of Ypd1p and derepression of Ssk1p. Finally, unphosphorylated Ssk1p binds to the MAPKKKs Ssk2p and Ssk22p, which autophosphorylate, and then can phosphorylate the MAPKK Pbs2p. Sln1p seems to dominate the kinetic response of the pathway while also ensuring its robustness by inducing high basal Hog1p expression counteracted by a fast-acting negative feedback to allow rapid pathway response [53]. Thus, this tightly tuned signaling branch allows rapid and sensitive responses to environmental changes.

Sho1p consists of four transmembrane domains and an SH3 domain. This domain permits the recruitment of molecular actors, notably the MAPKK Pbs2p, to the plasma membrane [54]. The upstream kinase Ste20p, the G-protein Cdc42p, and the MAPKKK Ste11p needed for the activation of the protein Pbs2p are also recruited to the membrane [55]. Since it is a transmembrane protein, Sho1p has long been considered an osmosensor [56]. However, recent studies suggest that Sho1p is more an anchor protein than a sensor for osmolarity [55]. Hkr1p and Msb2p, two mucin-like [57–60] proteins that form heterooligomeric complexes with Sho1p [58, 59] have recently been proposed as osmosensors of the SHO1 branch. Components of the SHO1 branch also take part in pseudohyphal development and mating, indicating that Sho1p might not have a specific role in osmosensing but a more general role related to cell shape measurement [61].

MAPKKKs of these two initiating branches induce the phosphorylation of the MAP kinase kinase Pbs2p on the conserved residues Ser514 and Thr518 [62]. Pbs2p is a cytoplasmic protein essential for the activation of Hog1p by dual phosphorylation on the conserved Thr174 and Tyr176 [62]. *PBS2* and *HOG1* are essential for osmoadaptation as null mutations in both genes induce osmosensitivity [23, 63]. Pbs2p also plays the role of a scaffold for the SHO1 branch [49, 54, 56, 64] by anchoring the different components, promoting signal propagation between proper protein partners and preventing improper cross-talk between the Pheromone pathway and the HOG pathway. Once Pbs2p phosphorylates Hog1p, Hog1PP translocates to the nucleus in a manner that is dependent upon the karyopherin Nmd5p [65]. Localization of Hog1p-GFP to the nucleus can be used as a reliable reporter of pathway activity.

## 4. Sequential Response after a Hyperosmotic Shock

**Figure 3:**
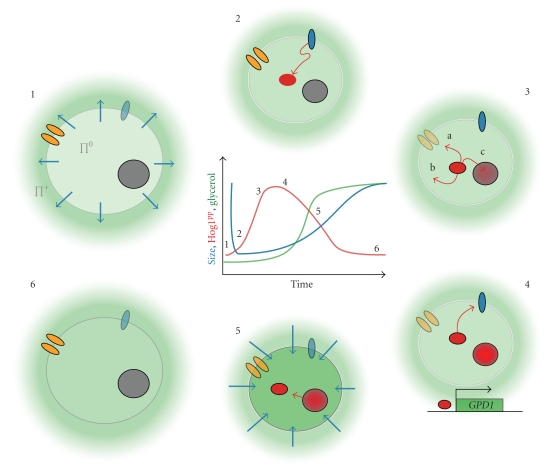
Sequential sketch of yeast adaptation to a hyperosmotic shock. The evolution with time of the size, phosphorylation of Hog1p, and internal concentration of glycerol are schematically represented in the center of the picture. (1) After an increase of the external osmolarity (green), a first mechanical response corresponds to a rapid loss of water (blue arrow). It leads to a decrease of the cell size and a loss of turgor pressure. (2) HOG osmosensors (blue) activate the pathway and eventually lead to the phosphorylation of Hog1p. (3) Hog1PP induces several processes: (a) Inactivation of the glycerol channel Fps1p preventing glycerol leakage; (b) direct or indirect activation of cytoplasmic actors, for example, 6-phosphofructo-2-kinase (Pfk2p) involved in glycerol synthesis; (c) translocation in the nucleus. Note that there are other targets of Hog1p such as Sic1p, Hsl1p, Nha1p, and Tok1p. (4) Nuclear Hog1PP induces a large transcriptional response. In particular, the gene GPD1 leading to glycerol synthesis is upregulated. Negative feedbacks (glycerol production, phosphorylation of Sho1p, etc.) allow inhibition of the pathway activity. (5) Increase of the internal glycerol leads to water influx and progressive cell size recovery while Hog1p is exported from the nucleus. (6) Pathway is off, and turgor pressure and cell size are restored. The cell is adapted to its new environment.

The activation of the Hog1p MAPK triggers several responses on different time-scales (Figure 3) [48]. A rapid nontranscriptional response in the cytoplasm corresponds to the closure of Fps1p [66] and the activation of several kinases (e.g., Rck2p [67], Pfk2p [68]). Fps1p belongs to the ubiquitous Major Intrinsic Protein (MIP) [69] family and is known to play a central role in yeast osmoadaptation by controlling both uptake and efflux of the osmolyte glycerol [70]. Importantly, Fps1p is gated by osmotic changes [66, 71]. Indeed, this channel protein is closed under hyperosmotic stress to enable intracellular accumulation of glycerol, whereas it is open under low-osmolarity conditions to allow for glycerol efflux. 

On a longer time scale, several minutes after an osmotic shock, Hog1p induces the modification of expression of nearly 600 genes [72–75]. This transcriptional response is driven by intermediate transcriptional factors: Hot1p, Sko1p, Smp1p, and Msn2/4p [37, 38, 74, 76, 77]. Importantly, Hog1p initiates glycerol biosynthesis via the transcriptional factor Hot1p [38]. Glycerol production is due to the expression of glycerol-3-phosphate dehydrogenase and glycerol-3-phosphatase. Both enzymes are encoded by two similar isogenes, *GPD1*, *GPD2* and *GPP1*, *GPP2,* respectively, [78, 79]. The accumulation of glycerol results in an increase of the internal osmolarity, leading to water influx and cell size recovery. Hot1p is also involved in regulating glycerol influx by inducing a strong and transient expression of *STL1*, which codes for a glycerol proton symporter located in the plasma membrane [80]. Hog1p is dephosphorylated and exported from the nucleus via the karyopherin Xpo1p [65] 20 to 30 minutes after an osmotic shock depending on the severity of the shock. This is concomitant with the onset of glycerol production and restoration of osmotic balance. Dephosphorylation of Hog1p is due to nuclear phosphatases. Phosphatases have a critical role in downregulation of MAPK proteins whose excessive activation can be lethal for the cell. In yeast, three classes of protein phosphatases are known to downregulate MAPK pathways. The dual specificity phosphatases (DSPs) dephosphorylate both phosphotyrosine (pY) and phosphothreonine (pT). The protein tyrosine phosphatases (PTPs) dephosphorylate only tyrosine residues. Finally, protein phosphatases type 2C (PTC) dephosphorylate threonine, serine, and sometimes tyrosine residues. For the HOG pathway, the serine-threonine phosphatases Ptc1p, Ptc2p, and Ptc3p act on both the Pbs2p (MAPKK) and Hog1p (MAPK), while the tyrosine phosphatases, Ptp2p and Ptp3p strictly control Hog1p [43, 44, 49]. Ptp2p is predominantly localized in the nucleus, Ptp3p in the cytoplasm, while the protein phosphatases types 2C are located both in the cytoplasm and in the nucleus. Simultaneous knockout of both *PTP2* and *PTC1* is lethal for the cell [45]. Deletion of *PTP3* induces overactivation of Hog1p but is not lethal because it predominantly acts on other MAPK proteins involved in the mating pathway.

## 5. Towards a Model of the HOG Pathway

Years of genetic and biochemical analysis have provided us with an extraordinarily precise description of the key players in the HOG pathway. What about the signaling dynamics of the pathway? How does the architecture determine the pathway's signal processing ability? Classic molecular biology experiments were based on step shock experiments with an osmotic agent, such as NaCl or sorbitol at various concentrations. Phosphorylation states of key proteins have been measured at different time points after a step shock at the population level, showing a transient increase of phosphorylation (lasting several minutes) concomitant with nuclear enrichment of Hog1p [81]. Nuclear cytoplasmic shuttling of Hog1p was also observed qualitatively, indicating a fast deactivation of the pathway when cells are returned to an isotonic environment [81]. Levels of gene expression have been measured at different timepoints after an osmotic shock using microarrays [73]. Although done with a low resolution in time compared to biophysical experiments, these measurements give an idea of the dynamics of the activation of the pathway. 

Based on such measurements, several models have been proposed to describe mathematically the HOG signaling pathway and more generally osmoadaptation in yeast [82]. The most comprehensive and the first integrative one is due to Klipp et al. [81]. Their model takes not only the HOG signaling cascade into account (only the SLN1 branch), but also includes a description for the metabolic production of glycerol, as well as an elementary gene expression model for the enzymes involved in glycerol production. The model also includes the closure of the membrane glycerol tranporter Fps1p and takes the dephosphorylation of nuclear Hog1p by Ptp2p into account. Most reactions in the model were described by the mass action rate law. The model consisted of 70 parameters, of which 24 had to be estimated. To estimate this number of parameters with the limited data available, the authors divided the model in modules and fitted them separately to data points. Their model reproduced accurately the transient response of the HOG pathway after a single hyperosmotic shock. This included the phosphorylation states of Hog1p and Pbs2p, as well as glycerol production and cell-size recovery. In addition, the model was able to correctly predict the effect of different mutations of proteins involved in the pathway. Mutants unable to produce glycerol (*gpd1Δ, gpd2Δ*) [83] or to close the Fps1p channel showed an increased duration of HOG activity. Mutants with an increased phosphatase Ptp2p activity showed a lower level of phosphorylated Hog1p but a similar period of HOG activity. 

Although very promising, such an approach is still extremely difficult to fine tune since it relies on many unknown parameters. Comparison of the model outputs to experimental data is crucial. To further constrain and test complex models one needs quantitative, time-resolved experiments at the single-cell level in response to complex input signals. 

As engineers do with electronic circuits and chips, a very powerful way to explore the dynamics of a given system is to observe its response to complex input signals. Such an approach lends itself to developing minimal models that capture the dynamical properties of the pathway, such as feedback loops and signal processing abilities, without taking into account all the details of the biochemical reactions. These approaches require designing experimental systems in which the extracellular environment can be quickly and precisely varied. We will now review the innovative methodologies that have been recently used to study single yeast cells in time varying environments. Then, we will review how those measurements have been integrated into minimal modeling to further study the dynamics of the HOG pathway.

## 6. Fast Control of the Chemical Environment of Single Cells

**Figure 4:**
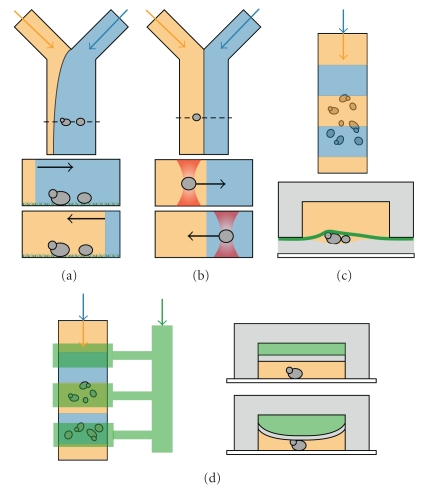
Different microfluidics techniques to control the chemical environment of single yeast cells while imaging them through microscopy. (a) Microfluidic system as described in Hersen et al. [11]. Yeast cells are fixed in the channel by the lectin protein Concanavalin A. One inlet is filled with an iso-osmotic media (blue) and the other with a hyperosmotic media (orange). By tightly controlling the pressure in each inlet, it is possible to create a periodic shock on the cells. (b) Optical tweezers system (red) as described by Eriksson et al. permits to control the cells position in the channel with two fluids flowing side by side [12]. (c) The system developed by Charvin et al. uses a dialysis membrane (green) to trap cells on top of a soft PDMS slice [13]. (d) Multilayer microfluidic device [14]. The top layer (green) is used to capture cells. By controlling the pressure inside this channel, cells can be optimally trapped while subjected to periodic shocks. The bottom layer is used to culture cells.

Several approaches, using microfluidics [8, 9, 84, 85], have been recently proposed to allow for a fast and reliable control of the chemical environment of yeast cells [7]. Hersen et al. [11] designed a fast binary switch to repeatedly change the environment of single yeast cells between two chemical conditions as fast as every second (Figure 4(a)). They used a Y-shaped flow chamber, 50 *μ*m high and 500 *μ*m wide, with two inlets. One inlet was filled with an isotonic medium, and the other with the same culture medium complemented with sorbitol to increase its osmolarity. At such small scales, flows are laminar and fluids do not mix but rather simply flow side by side. The lateral position of the fluids interface is set by the relative hydrostatic pressure—or the relative flux—of the two inlets. Changing this pressure difference displaces the interface laterally in less than a second. Yeast cells, previously fixed in the channel through concanavalin-A coating were then repeatedly switched from an isotonic to a hyperosmotic environment. An interesting alternative developed by Eriksson et al. [12] consists of moving the cells with optical tweezers (Figure 4(b)) rather than moving fluids over fixed cells. This strategy removes the potential influence of cell adhesion on signaling dynamics related to morphological changes, but at the cost of technological complexity. Also, such a strategy is very time consuming. Holographic tweezers—a sophisticated version of optical tweezers—can help to increase the number of cells that can be observed in real time [86]. Another strategy was proposed by Charvin et al. [13, 87]. Yeast cells are fixed between a permeable dialysis membrane and a cover slip coated with a very thin layer of soft PDMS (Poly-Di-MethylSiloxane). A channel is placed on top of the membrane and allows flow of fresh media and exchange within a few minutes. Nutrients and other chemicals can freely diffuse through the membrane. With this device, environmental exchange happens more slowly, but cells can grow over several generations in a monolayer simplifying their observation through microscopy. Indeed, Charvin et al. used it to force periodic expression of cyclins in yeast growing exponentially up to 8–10 generations.

More complex devices have been proposed, though they require a high degree of expertise to fabricate and manipulate. Bennet et al. [88] developed an environmental switcher capable of generating sinusoidal inputs. Their multilayer device was composed of a microchemostat, with a depth of 4 *μ*m to force yeast cells to grow in a monolayer, and a fluid mixer to generate complex time varying environmental signals for the cells in the chemostat chamber. They used this device, in a particularly elegant work, to revisit the wiring of the GAL system in yeast, by subjecting cells to sinusoidal inputs of carbon source over a range of frequencies. Taylor et al. [14] described a high throughput microfluidics single-cell imaging platform to study the dynamics of the pheromone response in yeast. They combined a fluidic multiplexer, an array of channels, and many sieve valves to trap cells and to control fluid delivery. They were able to perform simultaneous time lapse imaging of 256 chambers with 8 different genotypes with several dynamical inputs. Such a strategy, although very sophisticated, can enhance dramatically the quantity of data gathered to improve our knowledge and refine modeling of MAPK pathways in yeast [7].

## 7. New Insights from Coupling Complex Stimulus and Reverse Systems Engineering

Using such microfluidics strategies (Figure 4(a)), Hersen et al. studied the HOG pathway response to periodical osmotic stimulation over a range of frequencies. Interestingly, the HOG pathway acts as a low-pass filter, meaning that the output of the pathway (Hog1p nuclear localization) does not follow a fast varying input precisely, but rather integrates fast fluctuations over time. For wild-type strains, when the input signal varies slower than once every 200 s, Hog1p cytoplasmic—nuclear shuttling follows the input variations faithfully [11, 17]. However, when the input varies more rapidly than every 200 s, Hog1p nuclear translocation no longer follows the input faithfully, but instead integrates over the input fluctuations [11, 17]. This typical time is also the slowest time (or limiting step) of activation of the pathway although it was not possible from these experiments to point out which biochemical step was limiting. By genetic removal of one of the two branches, the contribution of each branch was also measured by Hersen et al., and it was found that the SHO1 branch is slower than the SLN1 branch by almost a factor two. The SHO1 branch was actually unable to integrate the too fast variations of the input whereas the SLN1 branch, when taken alone, was displaying a similar behavior than wild-type cells [11]. Those investigations clearly evidenced that the pathway can be turned off very quickly and repeatedly, suggesting the existence of several feedback loops acting on different timescales. 

**Figure 5:**
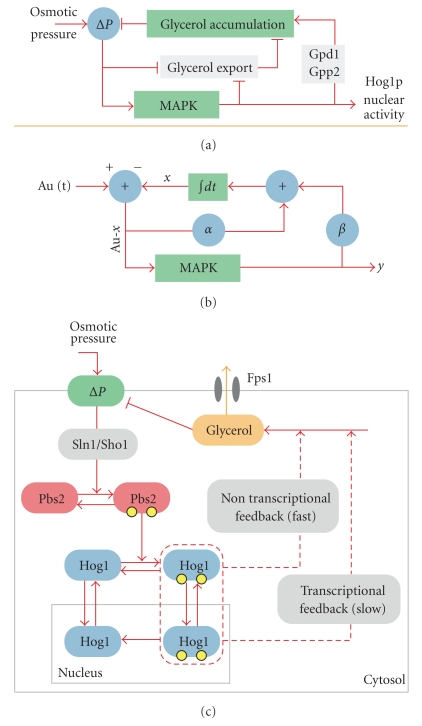
Schematic representation of the Hog pathway models of Mettetal et al. [10] and Zi et al. [15]. Pictures are redrawn from original figures of these papers. Top: (a) Diagrammatic representation of Mettetal's model. Au(t) represents the osmolarity applied at time *t* and the variables *x* and *y* can be identified with the intracellular glycerol concentration and the enrichment of Hog1 in the nucleus. The model contains a feedback depending on Hog1p (with strength *β*) and one, which is independent of Hog1p (strength *α*). The equations for this model read $\dot{y}=(A_{0}u-x)-\gamma y$ and $\dot{x}=\alpha (A_{0}u-x)+\beta y$. (b) The same model, interpreted in biological terms. The export of osmolytes is regulated by a mechanism, which does not depend on the MAPK pathway (e.g., closure of Fps1p) and by a mechanism depending on Hog1p activation. (c) Diagram of the model structure proposed by Zi et al. The model includes a simplified version of the MAPK pathway as well as two different feedbacks induced by activated Hog1p (a slow transcriptional and a fast nontranscriptional). Both of these feedbacks act by increasing the production of glycerol.

An attempt to decipher the dynamical aspects of these feedback loops has been done by Mettetal et al. [10], who also examined the response of the Hog1p nuclear localization in response to an oscillating input. They constructed, based on these frequency experiments, a simple predictive model, which was not based on biological knowledge (Figure 5(a)). Subsequently, they identified the two variables of their model with the intercellular osmolyte concentration and the phosphorylation state of Hog1p and concluded that the pathway contains a Hog1-dependent and a Hog1-independent feedback mechanism. By underexpressing Pbs2p, thereby reducing the sensitivity of the Hog1-response to the input, they were able to isolate the Hog1-independent feedback from the Hog1-dependent feedback. Based on this they concluded that the Hog1-dependent feedback is required for fast pathway inactivation. By inhibiting translation, they showed indeed that the slow transcriptional response triggered by Hog1p is only necessary for the adaptation to multiple osmotic shocks, while for a single osmotic shock faster nontranscriptional feedback mechanisms dominate the response. Their conclusion is in perfect agreement with recent experimental investigations showing that even cells with Hog1p anchored to the membrane present an increase of glycerol production after a hyperosmotic shock [89]. Although the details are not known, Hog1p directly or indirectly activates the 6-Phosphofructo-2-kinase (*PFK2*) [68] which leads to an increase production of glycerol through Gpd1p activity. 

Hao et al. also focused on rapid non-transcriptional feedback loops. First, they noticed that the response of the SHO1 branch is more transient than that of the SLN1 branch. Then, based on previous observations, they constructed three simple mathematical models, each describing another possible mechanism of HOG inactivation. One model was based on Hog1p mediating activation of a negative regulator (phosphatases), while the other two models focused on the negative control of a positive regulator. Analysis of the different models suggested a Hog1p-dependent feedback mechanism occurring early in the response. Their experimental analysis confirmed this and suggested that Hog1p acts negatively on Sho1p by phosphorylation, thereby implementing a direct negative feedback loop.

Muzzey et al. [18] followed a similar approach to study the feedback mechanisms within the pathway. They identified the transient activation of Hog1p with a feature called perfect adaptation, which states that the steady state output of the pathway does not depend on the strength of the osmotic shock. They argued that robust perfect adaptation requires at least one negative feedback loop containing an integrating component [90] and they analyzed the location of this integrator. They defined an integrating component as a dynamic variable whose rate of change does not depend on itself. They monitored multiple system quantities (cell volume, Hog1p, and glycerol) and used varied input waveforms to analyze the pathway. Similar to Hao et al. [16], they constructed different variants of a mathematical model, each with a different location of the integrating component. The authors found that the integral feedback property is Hog1p dependent and regulates glycerol uptake. 

More recently, Zi et al. [15] analyzed the experimental frequency response of the HOG pathway done by Hersen et al. and Mettetal et al. They constructed a minimal model that can reproduce the response of the pathway to oscillating inputs (Figure 5(b)) [15]. They defined a signal response gain, which is defined as the ratio of the integrated change of the output of the pathway to the integrated input change and represents a measurement for the efficiency of signal transduction. They concluded that yeast cells have optimized this signal response gain with respect to certain durations and frequencies of osmotic variations.

These different analyses have shown that the HOG signaling cascade can be described in a very simple and modular way with several feedback loops operating to deactivate the pathway: two operating on short time scales through Hog1p activity (Sho1p deactivation and glycerol production increase), and one depending on transcriptional activation of *GPD1*. The dynamics of the pathway was also precisely measured and it was shown that it behaves as a low-pass filter with a cutoff frequency, probably set by protein concentration. Interestingly, the SHO1 branch which is known to be involved in other cellular processes was shown to be slower in activating the Hog1p MAPK than the SLN1 branch. Finally, those approaches have provided us with an easily tractable mathematical model of the HOG pathway that can be efficiently coupled to detailed mechanistic models to study *in silico* the behavior of this MAPK pathway. Taken together, the coupling between mathematical modeling and experimental frequency analysis of the HOG pathway has given very important insights into the HOG pathway dynamics and more generally its functioning, demonstrating the interest of developing such strategies for studying signaling pathways in yeast.

## 8. Future Directions

Although the structure and the dynamics of the HOG signaling pathway are now well understood, several key points remain to be elucidated, the most elusive one being the mechanistic functioning of the two osmosensors, Sln1p and the Sho1p complex. Another important aspect of a better understanding of the HOG pathway is to integrate its behavior with other cellular processes. In particular, in 2000, Gasch et al. [73] compiled genome expression profiles of *S. cerevisiae *yeast subjected to several stress conditions and discovered that genes normally induced after a hyperosmotic shock are downregulated in response to a hypo-osmotic shock and vice versa. The CWI pathway is activated by hypo-osmotic stimulation [29], its physiological role being to reinforce the cell wall and prevent the cell from bursting. HOG and CWI do not share direct components but were seen to interact with each other [91, 92]. During cell growth both pathways may well be activated and deactivated within short intervals to balance between cell expansion and cell wall development. The Sln1p-dependent response regulator Skn7p [93, 94] could have a role in linking the cell-integrity pathway to the HOG pathway. Skn7p also interacts with Rho1p an upstream component of the CWI pathway. The evidence that Skn7p is apparently controlled by sensors of both the HOG pathway and the cell-integrity pathway makes Skn7p an excellent candidate for a regulator that coordinates osmoregulation and cell wall biogenesis [23, 93, 94]. More work is needed to better understand the putative role of Skn7p in coordinating different aspects of turgor pressure control and cell surface assembly. Using minimal models and fluctuating environments to activate periodically the CWI and/or the HOG pathway is one interesting way to explore their interactions. Similarly, it is known that the HOG pathway and the Pheromone pathway can interact [95–98]. For example, a *hog1Δ *strain will respond to a hyperosmotic shock by activating the response to pheromone pathway. Again, the dynamics of such cross-talk has not been intensely studied. Performing time varying inputs with both pheromone and hyperosmotic medium will provide invaluable experimental data to probe for the dynamical aspects of cross-talk between MAPK in yeast. 

Since MAPKs pathways are highly conserved from yeast to mammalian cells, it would be interesting to test higher eukaryotic cells, in single cell experiments, for similar system level properties. Although more difficult to implement than for yeast cells, microfluidic technics can also be used to control the external environments of mammalian cells both in time and space. Transposing the approaches described here to mammalian cells will probably give further insights in their signaling pathways dynamics.

## 9. Conclusion

Since its initial discovery in 1993 [24], extensive molecular and genetic research has uncovered the molecular actors, interactions, and functions of the components in the HOG signaling pathway. However, these methods are limited in that one cannot predict the behavior of a complex system from the analysis of isolated components. Understanding of the entire system requires the use of novel techniques borrowed from engineering, physics, and mathematics. Microfluidic technologies combined with live-cell microscopy have allowed the use of temporally complex stimuli to interrogate pathway function. Kinetic information obtained through biochemistry combined with knowledge of the molecular components has allowed for complex quantitative models of the HOG pathway to be constructed. These models in turn provide experimentally testable predictions about pathway behavior and function. Simple “black-box” models designed to mimic only key components of the pathway have proven useful for understanding specific phenomena. Thus, genetic and biochemical data combined with novel experimental approaches and modeling have allowed for the prediction of the dynamics and systems-level properties of HOG pathway signaling processes. These techniques are easily extended to other signaling pathways of interests with the final goal being to understand the relationships between structure, kinetics, and dynamics at the systems-level in complex biological networks.

## Figures and Tables

**Table 1:** The MAPK pathways in *S. cerevisiae. *The morphological adaptation corresponds to the cell behavior in response to each specific signaling input. The major molecular actors for each pathway are indicated below. Spore cell wall assembly during sporulation is another morphogenetic process driven by a MAPK protein (Smk1p), but with little knowledge on the other proteins involved and the structure of the pathway.